# Complications of Pessaries Amenable to Surgical Correction: Two Case Reports and a Systematic Review of the Literature

**DOI:** 10.3390/jpm13071056

**Published:** 2023-06-27

**Authors:** Laura Calles Sastre, Belén Almoguera Pérez-Cejuela, Augusto Pereira Sánchez, Sofía Herrero Gámiz, Javier F. Magrina, Mar Ríos Vallejo, Tirso Pérez Medina

**Affiliations:** 1 Department of Gynecologic Surgery, Puerta de Hierro University Hospital, Majadahonda, 28222 Madrid, Spain; almoguerabelen@gmail.com (B.A.P.-C.); augusto.pereira@salud.madrid.org (A.P.S.); sofiaherrerogamiz@gmail.com (S.H.G.); mar.rios.vallejo@gmail.com (M.R.V.); tirsoperezmedina@gmail.com (T.P.M.); 2 Department of Medical and Surgical Gynecology, Mayo Clinic Hospital, Phoenix, AZ 85054, USA; jmagrina@mayo.edu

**Keywords:** genital prolapse, pessary, complications, surgical complication, fistula, incarceration, cancer

## Abstract

Background: Forty percent of women will experience prolapse in their lifetime. Vaginal pessaries are considered the first line of treatment in selected patients. Major complications of vaginal pessaries rarely occur. Methods: PubMed and Embase were searched from 1961 to 2022 for major complications of vaginal pessaries using Medical Subject Headings (MeSH) and free-text terms. The keywords were pessary or pessaries and: vaginal discharge, incontinence, entrapment, urinary infections, fistula, complications, and vaginal infection. The exclusion criteria were other languages than English, pregnancy, complications without a prior history of pessary placement, pessaries unregistered for clinical practice (herbal pessaries), or male patients. The extracted data included symptoms, findings upon examination, infection, type of complication, extragenital symptoms, and treatment. Results: We identified 1874 abstracts and full text articles; 54 were assessed for eligibility and 49 met the inclusion criteria. These 49 studies included data from 66 patients with pessary complications amenable to surgical correction. Clavien–Dindo classification was used to grade the complications. Most patients presented with vaginal symptoms such as bleeding, discharge, or ulceration. The most frequent complications were pessary incarceration and fistulas. Surgical treatment included removal of the pessary under local or general anesthesia, fistula repair, hysterectomy and vaginal repair, and the management of bleeding. Conclusions: Pessaries are a reasonable and durable treatment for pelvic organ prolapse. Complications are rare. Routine follow-ups are necessary. The ideal patient candidate must be able to remove and reintroduce their pessary on a regular basis; if not, this must be performed by a healthcare worker at regular intervals.

## 1. Introduction

Pelvic organ prolapse (POP) is prevalent among older women. Milder stages of prolapse, cranial to the hymen, are common and usually symptomless. A specific symptom is a bulge outside the vagina. Functional symptoms from the bladder, bowel, and sexual life frequently coexist without a known cause/effect relationship with prolapse. Prolapse should be measured using the validated internationally approved pelvic organ prolapse quantification (POPQ) system, which uses the three vaginal compartments and three prolapse levels [1].

The recognition of prolapse can be traced back to Egypt in 1500 BC, and pessary use was recorded by Hippocrates in 400 BC. The word “pessary” is derived from the Greek word “peso”—an oval stone. Its probably originates from the use of oval stones or olive pits inserted into the uterus in saddle camels to prevent conception during long desert journeys [2]. A variety of pessary devices have been described over time [3]. Nowadays, most pessaries are made of silicone and are ring-type pessaries with or without central support, Gellhorn pessaries, and donut pessaries. The ring with central support and the Gellhorn pessary are the most frequently used and appear equally effective in relieving symptoms of genital prolapse and voiding dysfunction [4].

Pessaries have different uses, such as: conservative treatment for pelvic organ prolapse (in those patients who wish to avoid or postpone surgery), identifying occult stress urinary incontinence, the prevention of progressive prolapse, and the diagnostic assessment and prediction of surgical outcomes. Contraindications to pessary use are infections (vaginitis and pelvic inflammatory disease), latex sensitivity (only for latex devices), non-compliance, and lack of follow up.

The variety of shapes and sizes available enables individual fitting.

A 2004 Cochrane review of pessary use for POP, updated in 2013 [4,5,6,7], found only one randomized controlled trial examining the efficacy of pessary use. The aim of this randomized crossover trial was to compare symptom relief and change in quality of life for patients using the ring with support and Gellhorn pessaries [4]. Complications were described as rare, and there was no consensus on complication management. Furthermore, there was no reference to complication severity grading.

In 2015, Abdulaziz et al. carried out an integrative review of reported complications related to pessary use, and classified them according to a standardized severity scale (the Clavien–Dindo complication severity grading system) [8,9,10].

In 2021, Miranda Varella et al., reported a case series and conducted a literature review of rectovaginal fistulas (RVFs) induced by or developing shortly after the use of a pessary. There were a total of 23 instances of RVFs in the 17 studies. The review emphasized the possibility of RVFs associated with pessary use [11].

The objective of this report and literature review is to provide a recent evaluation of the serious complications arising from the use of pessaries requiring surgical repair.

### 1.1. Case Report 1

An 82-year-old woman (gravida 4, para 4) with a grade 3 cystocele and grade 2 hysterocele underwent her routine 6-monthly pessary replacement. She had been using a 75 mm PVC pessary ring for 6 years without complaints. During physical examination for pessary replacement, the pessary was firmly adhered to the vaginal fundus with two firm synechiae between the anterior and posterior aspects of the vagina. There were two attempts to remove the pessary, and both failed due to pain, so a vaginoscopy was scheduled in an office setting.

Office vaginoscopy was carried out using a rigid continuous-flow hysteroscope (Storz, Bettocchi); the optical system was 2.9 mm and the working channel was 5fr. To perform the procedure, the distension medium chosen was saline; a mechanical pump was used to control the instillation flow and intrauterine pressure, maintaining an average pressure of 70 mmHg throughout the procedure. Cold semi-rigid scissors were used to release the adhesions, and the pessary was then removed.

Hysteroscopy is a highly advanced and modern medical procedure that has proven to be highly effective in diagnosing and treating uterine pathology with minimal invasiveness. The vaginoscopy technique allows surgeons to see and treat vaginal pathology during the same procedure. This technique involves using a hysteroscope to visualize the vaginal canal and identify any abnormalities that may be present, such as polyps, cysts, or lesions.

In addition, the vaginoscopy technique is a less uncomfortable and invasive procedure for patients since it eliminates the need for a vaginal speculum, which can cause discomfort and pain for some patients. Furthermore, this technique can also be performed without the need for local anesthesia, further reducing the discomfort and invasiveness of the procedure.

In the case described, the vaginoscopy technique was used to release a pessary that was causing discomfort to the patient. (Supplementary Video S1) The use of cold semi-rigid scissors to release the adhesions was a safe and effective method for this purpose. Adhesions are bands of scar tissue that can form between tissues or organs. Therefore, just as hysteroscopy is an important tool for the diagnosis and treatment of uterine adhesions, vaginoscopy is equally important for vaginal adhesions.

### 1.2. Case Report 2

An 83-year-old woman (gravida 3, para 3) presented to our tertiary-care clinic with a longstanding history of uterine prolapse and year-long thick and malodorous vaginal discharge. Approximately 3 years earlier, she began using a pessary after an external gynecologist offered her a choice of nonsurgical or surgical options for symptomatic uterine prolapse. At presentation to our clinic, she was using a silicone ring pessary with a diaphragm, which was reportedly removed, cleaned, and reinserted monthly by her primary gynecologist.

The patient reported missing only about four nonconsecutive gynecologic appointments during the past 3 years because of illness in herself or her husband. With no warning, the vaginal discharge started, followed about 2 months later by spontaneous stool passage through the vagina.

About 9 months before presenting to our clinic for definitive surgical treatment, the patient was diagnosed with a rectovaginal fistula by her external physician. Immediate surgical treatment was deferred until inflammation decreased in the surrounding tissue. The patient said she had never taken hormone replacement therapy, either vaginally or orally. She reported no history of urinary incontinence, inflammatory bowel disease, diverticulitis, or perirectal infection. She had no history of third- or fourth-degree perineal lacerations from childbirth and no prior gynecologic or prolapse repair procedures.

Physical examination showed the pessary in the correct position. After its removal, a large 4 × 3 cm rectovaginal fistula was observed in the midportion of the vagina where the pessary and posterior vaginal mucosa had been in contact. The rectovaginal septum was markedly thinned, and a grade 4 uterovaginal prolapse was noted. The external anal sphincter and perineal body appeared intact.

The patient elected to undergo concomitant surgical correction of the uterovaginal prolapse and the rectovaginal fistula. Bowel preparation included polyethylene glycol electrolyte solution the night before the procedure and two enemas the next morning. Upon preoperative admission, the patient received cefotetan disodium for broad-spectrum antibiotic prophylaxis. The surgery included a vaginal hysterectomy, bilateral salpingo-oophorectomy, combined anterior and posterior colporrhaphy, uterosacral ligament suspension, and rectovaginal fistula repair.

Intraoperatively, the fistula was easily identified. The fistulous opening was excised, and the edges of the rectal wall freshened by excising the fistula opening to reach healthy rectal mucosa. The rectal wall was closed in a first continuous submucosal layer, a second layer of interrupted imbricating sutures, and a superimposing third layer of reinforcement with perirectal tissue, all using 3-0 polyglactin 910 sutures. The posterior vaginal wall and rectovaginal fascia were closed over the repair, and the pubococcygeus muscles approximated to the midline in the lower third of the vagina at the level of the perineum, which was closed with a subcuticular stitch using 4-0 polyglactin 910.

Postoperatively, the patient received total parenteral nutrition for 10 days. Once she tolerated a regular diet, she was discharged. At 8-week follow-up, she had no recurrence of fecal or prolapse symptoms.

## 2. Materials and Methods

### 2.1. Search Strategy

**Figure 1:**
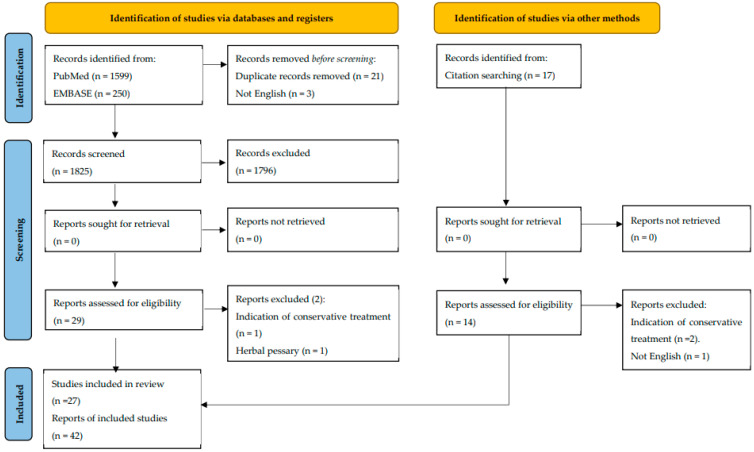
Flow diagram of study search and systematic review.

A systematic review was performed via PubMed and EMBASE respecting PRISMA guidelines (Preferred Reporting Items for Systematic Reviews and Meta-Analyses) [10] (Figure 1). Search terms were used as free terms and as Medical Subject Headings (MeSH) or Emtree terms (indexed on Pubmed or Embase from the years 1961 to 2022 inclusive). The following free terms were used on all databases: “pessary” OR “pessaries”, AND “vaginal discharge”, “incontinence”, “entrapment”, “urinary infection”, “fistula”, “incarceration”, “complications”, “vaginal infection”, “neglected”, “bowel”. The MeSH terms were used: “pessary OR pessaries”, AND “vaginal discharge”, “incontinence”, “entrapment”, “urinary infections”, “fistula”, “complications” OR “vaginal infection”. Hand-searching of citations was carried out on case series and reviewed studies to allow us to identify references that might have been missed in previous searches to prevent missing relevant information.

### 2.2. Selection Criteria

The databases PubMed and EMBASE were searched for articles dating from 1961 to 2022 inclusive. The selection criteria taken into consideration were female patients with major pessary complications implying first-line invasive treatment (surgical with local/general anesthesia), endoscopy, or major surgery (laparotomy). In this group were also included patients who had benefited from surgical treatment as a solution but not due to comorbidities. Articles were included only if they described clinical cases or case series of patients with vaginal pessaries with complications that were considered serious, in the English language, and that had an abstract. The exclusion criteria taken into consideration were: publications in a non-English language, pregnancy or fertility-related issues, fistulas or other complications without a prior history of pessary placement, pessaries not indicated for the treatment of genital prolapse (herbal pessary), or male patients.

### 2.3. Data Collection and Analysis

All articles were screened on the basis of title and abstract. The following data were extracted: title, author, year, journal, number of cases, age, clinical evolution, the initial presentation of symptoms, vaginal symptoms, vaginal entrapment or incarceration, presence of fistula, pelvic infection, urologic symptoms or any extragenital symptoms, examination findings, diagnosis delay, type of treatment, outcome, month of pessary insertion, total time of pessary use, and patient status.

All data were included in an EXCEL spreadsheet, and the final selection of articles was downloaded for full review; any disagreement was resolved via discussion.

All retrieved articles were case reports with or without literature reviews; due to the high heterogeneity of data obtained, a descriptive narrative review was planned instead of a meta-analysis. The risk of bias was minimized by adhering to the PRISMA statement. In addition, we created a flow diagram of the study search and systematic review with reasons for studies being excluded, removed, or not retrieved, using the PRISMA guidelines [12].

All data included in the study (in total, 66 patients from 49 studies) were compiled in a table according to patients with major complications of pessaries at the time.

### 2.4. Definitions

In order to unify the criteria for our study, we considered only grade III complications or higher according the Clavien–Dindo (CD) classification [10].

The CD classification consisted of 4 severity grades with several subgroups: The classification is presented in Table 1.

We included grade III complications or higher according to the CD classification. Grade III complications require surgical, endoscopic, or radiological intervention; grade IIIa intervention is not under general anesthesia; grade IIIb intervention is under general anesthesia; grade IV involves a life-threatening complication (including central nervous system complications) requiring IC/ICU management; grade IVa involves single-organ dysfunction (including dialysis); and grade IVb involves multi-organ dysfunction. Those patients who were candidates for surgical treatment but, due to their basal state, decided to undergo it with conservative management, were classified in a similar grade to candidates who underwent surgical treatment in the CD classification.

The time of pessary insertion was measured in months from the insertion until the initial presentation of symptoms, and total time of pessary use was measured in years.

A neglected pessary was defined as a pessary that has been left in place for an extended period without proper care or maintenance.

Pessary incarceration was defined as entrapment of the pessary via fixed vaginal adhesions.

Vesicovaginal (VVFs) and rectovaginal fistulas (RVFs) were defined as abnormal communication of the vaginal epithelium with the bladder epithelium or with the wall of the rectum, respectively.

## 3. Results

### 3.1. Main Findings

We identified 1874 abstracts and full text articles, and among them, 49 articles, with a total of 66 patients, met the inclusion criteria (19 PubMed, 22 hand-recruited, 8 Embase).

The number of patients under each grade of complication was as follows: 3a, 4 patients; 3b, 51 patients; 4b, 7 patients; and grade 5, 2 patients. Four patients initially classified as having grade 3 complications but were not ultimately treated.

### 3.2. Characteristics of the Patients

The mean age of the published patients was 78.8 (median 81; range 48; SD 10.7).

The mean time from the last pessary replacement to the complication, expressed in months, was 63.2, and the mean total time of pessary use, since the first insertion to the complication, expressed in years, was 11.6.

The most frequent major complication, present in 55 patients, was vaginal symptoms (80.9%). The most frequent vaginal symptoms were discharge (21 patients) and bleeding (17 patients).

The most frequent major complications found were fistulas (35), both vesicovaginal and rectovaginal, and incarceration of the pessary (20); migration of the pessary to the abdominal cavity through a fistula was also described in one previously hysterectomized patient, and a migration to the uterine cavity. In this review, nine cases of carcinoma were found and one case of profuse bleeding that required surgical treatment to control hemorrhage.

Regarding the care and follow-up of the patients, out of the 68 cases reviewed with major complications, 45 (66.2%) were neglected, 21 (30.9%) were not neglected, and data could not be obtained for 2 patients.

### 3.3. Diagnosis

To diagnose the different complications of pessaries, it may be necessary, from a simple detailed physical examination, as in the case of incarcerated pessaries, to perform different imaging tests, such as MRI or CT, as in the case of fistulas.

### 3.4. Therapy

The surgical treatments for these complications included:

In cases of incarceration of the pessary, surgical removal under local or general anesthesia is needed. The adhesions that incarcerate a pessary can be cut using scissors or a cold scalpel or, as described in the case we present, via endoscopy.

Treatment for a rectovaginal fistula depends on its cause, size, location, and effect on the surrounding tissues.

In some cases of vesicovaginal fistula, a Latzko operation was required to repair the fistula; it consists of a classical technique for vesicovaginal fistula repair using a vaginal approach. As an outpatient procedure with minimal morbidity and low cost, the Latzko operation is a high-value procedure. Other cases required the performance of ureteroneocystostomy with psoas hitch.

In several cases, surgical treatment was required to control hemorrhage. In two cases, major surgical interventions were necessary, including intestinal resections to correct the fistula with colostomy in one case, and ileostomy in the other. After the resolution of the complications, surgery was continued to correct the prolapse, such as vaginal or abdominal hysterectomy or LeFort colpocleisis (Table 2).

## 4. Discussion

We present a systematic review of the major complications of pessary amenable to surgical correction and two case reports in which surgeries were necessary to solve their complications. 

The use of pessaries is more common in older patients, especially those who have a high surgical risk or comorbidities that make them unsuitable for surgical intervention. 

The average age of patients using pessaries is typically higher because the prevalence of pelvic organ prolapses and urinary incontinence increases with age (78.8 years). Additionally, older patients may be more likely to have medical conditions that make them unsuitable for surgery, such as cardiovascular disease, diabetes, or respiratory problems, and this makes them more vulnerable in case of complications.

The frequency of complications is highly variable between different publications. The most frequent symptoms were vaginal symptoms (80.9%). The most frequent vaginal symptoms were bleeding and discharge. Dangerous complications, including death, are very rare but have been described, particularly if the pessary is neglected [62].

We included grade III complications or higher, according to the CD grades, for complications that occurred because of pessary use; 51 patients met the criteria for 3b in the CD classification.

The most frequent major complications found were incarceration of the pessary (20) and fistulas, both vesicovaginal and rectovaginal (35).

Pessary incarceration is an entrapment of the pessary via fixed adhesions in the vagina. There are several theories about how these adhesions come to be formed; erosions and ulcerations of the vaginal epithelium can give rise to these adhesions, which could trap the pessary [26]. Once again, we stress the importance of careful monitoring of these patients. In the case presented, due to close patient follow-up, the adhesions were found to be loose and could be resected using vaginoscopy in the office without the need for general anesthesia. Overall, hysteroscopy with vaginoscopy is a highly advanced and effective medical procedure that has revolutionized the field of gynecology. Its minimal invasiveness and high accuracy in diagnosis, and the ability to perform operative procedures in an ambulatory setting, make it a highly valuable tool for the treatment of various gynecological conditions. 

VVFs and RVFs, although uncommon, are among the most serious complications of neglected pessaries [63]. They are defined as abnormal communication of the vaginal epithelium with the bladder epithelium or with the wall of the rectum, respectively. Fistula formation may also be associated with fecal impaction, hydronephrosis, and urosepsis [64], although as we mentioned before, serious complications are rare. In case reports describing VVFs, bowel fistulae, and incarcerated pessaries, 91% were correlated with neglected pessaries [34]. These complications have physical alterations that require surgical treatment for their resolution, as the appearance of fistulas is associated with symptoms such as urinary or fecal incontinence, bleeding or pain that can even cause psychological alterations, and quality of life in the patient.

In the case that we present (case report 2) the patient had only missed four non-consecutive appointments. The follow-up allowed for the diagnosis of the complication and its satisfactory surgical resolution. Even with proper fitting and frequent examinations, complications may still occur. Patients should therefore be informed about this possibility, especially when regular maintenance may be neglected.

There are some reports that implicate pessaries as a causal mechanism for both vaginal and cervical cancer, although there is no scientific evidence in the literature of a link between pessary use and cancer. There are two possible mechanisms proposed to explain this relationship. One would be that chronic inflammation in association with viral infections can predispose patients to such cancers as the tumors appear at the site of pessary placement [64]. The other mechanism proposed includes the generation of metaplastic and subsequent dysplastic changes in the squamous mucosa [13,64,65,66].

However, as highlighted in this systematic review, the use of pessaries is not without risk. Complications related to pessary use can range from mild symptoms, such as vaginal discharge and odor, to more serious complications, such as vesicovaginal and rectovaginal fistulas, erosion, ulceration, and even death. 

One of the most concerning aspects of this review is the fact that dangerous complications can occur, particularly if the pessary is neglected (66.2%). The authors note that although rare, death has resulted from pessary use in some instances. This is a sobering reminder of the importance of proper monitoring and follow-up care for patients using pessaries, especially for older women who may be at higher risk of complications. In addition to the risk of death, the review also highlights the significant impact that pessary-related complications can have on the quality of life of affected women.

For example, vesicovaginal and rectovaginal fistulas can cause abnormal communication between the vaginal epithelium and the bladder or rectum, leading to symptoms such as urinary or fecal incontinence. These symptoms can be particularly distressing for older women, who may already be dealing with other health issues. 

It is also worth noting that this review highlights the lack of objective classification of severity in pessary-related complications. This can make it difficult for healthcare providers to accurately assess the risks and benefits of pessary use and enable patients to provide informed consent. It is important for providers to have a clear understanding of the potential complications of pessary use and to communicate these risks effectively to patients, particularly older women who may be more vulnerable to these complications. 

Although less frequent, as evident from the review, there are reported cases of fistula-type complications after the short-term use of a pessary.

There is a higher risk in obese patients, possibly due to increased pressure exerted by the pessary at a specific point in the vagina. Additionally, patients with enteroceles and weakened vaginal walls may also be at increased risk. This risk may be present in previously hysterectomized patients who have developed complications such as postoperative infection, leading to impaired healing of the vaginal vault [25].

The main limitations of this review are primarily related to the significant heterogeneity found in the publications. These limitations include the data collected, the lack of clear definitions in the reviewed manuscripts (the method of diagnosis of the complication, the exact time of use of the pessary, and the characteristics of the patients), and follow-up protocols. In addition, we acknowledge the exclusion of patients from two articles written in a non-English language (French and German). In this context, we consider that a meta-analytic approach is not feasible and could even contain biases. The main strength of our study is that it provides a better understanding of the possible complications in the use of pessaries, since due to their rarity, they are not well known and can go unnoticed or undergo late diagnosis, and that it points out the need of future investigation. Future research should take into account the limitations described above.

## 5. Conclusions

While pessaries can be a valuable non-surgical option for the treatment of pelvic organ prolapse, the risks associated with their use cannot be ignored. This review emphasizes the need for proper monitoring, follow-up care, and informed consent to ensure the safety and well-being of patients using pessaries, especially older women who may be at higher risk of complications. Healthcare providers must weigh the benefits and risks of pessary use carefully and take a personalized approach to treatment to provide the best possible outcomes for their patients.

As there are only published cases or case series, there is not enough evidence to be able to establish the real risks inherent in the use of pessaries, so it would be interesting to propose clinical trials to obtain more scientific evidence.

## Figures and Tables

**Table 1:** Classification of surgical complications and literature review of complications according to Clavien–Dindo classification.

Type	Clavien–Dindo Classification	N
**Grade I**	Any deviation from the normal postoperative course without the need for pharmacological treatment or surgical, endoscopic, and radiological interventions.	
**Grade II**	Requiring pharmacological treatment with drugs other than those allowed for grade I complications. Blood transfusions and total parenteral nutrition are also included.	
**Grade III**	Requiring surgical, endoscopic, or radiological intervention.	4 (no treatment)
**IIIa**	Intervention not under general anesthesia.	4
**IIIb**	Intervention under general anesthesia.	51
**Grade IV**	Life-threatening complication (including CNS complications: brain hemorrhage, ischemic stroke, subarachnoidal bleeding, but excluding transient ischemic attacks (TIA)). Requiring Intermediate care/Intensive care unit management.	
**IVa**	Single-organ dysfunction (including dialysis).	
**IVb**	Multi-organ dysfunction.	7
**Grade V**	Death of a patient.	2

Abbreviations: N = number of registered complications in systematic review.

**Table 2:** Literature review of patients with pessary complications.

Authors	Age, Yr	Initial Presentation of Symptoms	Vaginal Symptoms	Vaginal Entrapment	Extragenital Symptoms	Presence of Fistula	Examination Findings	Pessary Insertion before Complication, Mo	Total Pessary Time, Yr	Neglected Pessary	Treatment
Russel K et al., 1961 [13]	77	Vaginal bleeding	Yes	Yes	No	Yes	V-VF	216	18	Yes	Failed surgical VVF closure
Russel K et al., 1961 [13]	55	Vaginal bleeding	Yes	Yes	No	No	Vaginal cancer	108	9	Yes	-
Russel K et al., 1961 [13]	74	Vaginal bleeding	Yes	No	No	No	Vaginal cancer	-	40	Yes	-
Russel K et al., 1961 [13]	66	Vaginal bleeding	Yes	No	No	No	Vaginal cancer	96	10	Yes	-
Russel K et al., 1961 [13]	84	Vaginal bleeding	Yes	No	No	No	Vaginal cancer	3	8	No	-
Russel K et al., 1961 [13]	75	Vaginal bleeding	Yes	No	No	No	Vaginal cancer	2	20	No	-
Russel K et al., 1961 [13]	70	Vaginal discharge	Yes	Yes	No	No	Infection and CPI	9	-	No	Surgical removal under GA and LeFort
Russel K et al., 1961 [13]	78	Vaginal discharge	Yes	Yes	No	No	Embedded pessary	312	26	Yes	Surgical removal
Russel K et al., 1961 [13]	77	Vaginal discharge	Yes	No	No	No	Vaginal cancer	0.5	50	-	-
Summers et al., 1971 [14]	90	Hematuria	No	No	Yes	No	Embedded pessary	684	57	Yes	Pessary removal under GA
Thornton et Harrison, 1977 [15]	-	Vaginal wall ulceration	Yes	Yes	No	No	Vaginal ulceration	4	0.33	-	Surgical excision (GA)
Goldstein et al., 1990 [16]	82	Urinary leakage	Yes	No	Yes	Yes	V-VF	29	2.4	Yes	Fistula repair
Ott et al., 1993 [17]	72	Abdominal pain and transvaginal bowel prolapse	Yes	No	Yes	No	Perforation of the vaginal stump and terminal ileum incarceration	60	5	Yes	Surgery
Dasgupta et al., 1996 [18]	81	Lethargy	No	No	No	No	Uremia, ureter obstruction	6	5	No	Pessary removal, double-J stent
Duncan et al., 1997 [19]	85	Fever, abdominal pain, confusion, vomiting	No	No	Yes	No	Ureterovesical junction obstruction caused by the pessary	-	-	Yes	Pessary removal and French double-J ureteral stent
Grody et al., 1999 [20]	98	Involuntary loss of urine	Yes	No	Yes	Yes	V-VF	-	18	Yes	Surgery repair (Schuchardt’s incisions)
Cumming et al., 2000 [21]	74	Profuse vaginal discharge and pyrexia	Yes	No	Yes	Yes	V-VF	2d	0.0054	No	Conservative
Osei Kankam et al., 2002 [22]	88	Constipation, lethargy, weight loss	No	No	Yes	Yes	R-VF	-	-	Yes	Failure of fistula closure (deceased)
Chou et al., 2003 [23]	83	Urinary incontinence	Yes	Yes	Yes	No	VPI	120	10	Yes	Surgical removal
Wheeler et al., 2004 [24]	75	Abdominal pain, vomiting	No	No	Yes	No	Edematous pelvis, pus- covered shelf pessary	6	3	Yes	Laparotomy, vaginal removal of pessary
Sinha et al., 2004 [25]	56	Abdominal pain, mass extruding vaginally	Yes	No	No	Yes	Spontaneous vaginal evisceration of small intestine	1	83	No	Laparotomy
Liang et al., 2004 [26]	70	Nocturia	Yes	Yes	No	No	VPI	42	4	Yes	Surgical removal (GA)
Tse Ka Yu et al., 2004 [27]	51	Vaginal discharge	Yes	Yes	No	No	Ulceration and CPI	4	7	No	Pessary removal
Hanavadi et al., 2004 [28]	88	Vaginal discharge	Yes	No	Yes	Yes	R-VF	-	-	Yes	Surgical (colostomy)
Nallendran et al., 2006 [29]	86	Difficulty of pessary removal	No	Yes	No	No	Embedded pessary	120	10	Yes	Pessary removal under GA and hysterectomy
Popli et al., 2007 [30]	85	Urinary incontinence, vaginal pain	Yes	No	Yes	Yes	V-VF	4	Long time	No	Surgical fistula and prolapse repair
Luyer et al., 2007 [31]	81	Abdominal pain	No	No	Yes	No	Cecal rupture due to a dislocated vaginal pessary	-	-	Yes	Removal, fecal peritonitis led to overwhelming sepsis (deceased)
Kaaki et Sangeeta, 2007 [32]	84	Urinary vaginal leakage	Yes	No	No	Yes	V-VF	6	12	No	Latzko fistula repair and LeFort colpocleisis
Esin et Harmanli, 2008 [33]	85	Urinary incontinence	Yes	No	No	Yes	V-VF	120	10	-	Fistula repair and LeFort colpocleisis
Esin et Harmanli, 2008 [33]	93	Urinary incontinence	Yes	No	Yes	Yes	V-VF	10	4	-	Fistula repair and LeFort colpocleisis
E. Arias et al., 2008 [34]	89	Urinary incontinence	Yes	No	No	Yes	V-VF, pessary in bladder	36	3	Yes	Surgical removal (Schuchardt’s incisions)
Powers et al., 2008 [35]	70	Asymptomatic	Yes	Yes	No	Yes	R-VF	36	3	Yes	Transanal removal and surgical repair
Lim et Collaris, 2008 [36]	62	Asymptomatic (follow up)	No	No	No	Yes	Pessary in abdominal cavity	Follow up	0.048	No	Surgical removal (GA)
Ray et al., 2006 [37]	93	Urinary frequency, abdominal pain	Yes	No	Yes	Yes	V-VF	5	-	No	Spontaneous closure
Siddiqui et al., 2011 [38]	79	Blood-stained vaginal discharge and abdominal pain	Yes	No	Yes	Yes	Spontaneous vaginal evisceration	-	Long time	-	Vaginal hysterectomy and defect closing
Yong et al., 2011 [39]	83	Vaginal discharge	Yes	Yes	-	Yes	R-VF	60	5	Yes	Transvaginal repair and graft interposition
Dasari et Sagili, 2012 [40]	60	Purulent discharge and urinary stress incontinence	Yes	Yes	No	Yes	Metal ring pessary embedded in vagina	360	30	Yes	Surgical removal (GA)
Andrikopoulou et Lazarou, 2015 [41]	91	Vaginal bleeding	Yes	Yes	No	No	Blood vaginal accumulation due to pessary impaction	168	14	Yes	Surgical removal (GA)
Ghanbari et al., 2019 [42]	84	Vaginal bleeding	Yes	Yes	No	No	VPI	108	10	Yes	Surgical removal
Asumpinwong et al., 2019 [43]	77	Vaginal discharge	Yes	No	No	No	Doughnut pessary located in the uterine cavity	84	Long time	Yes	Abdominal hysterectomy due to failure of vaginal removal
Gaigbe-Togbe et al., 2020 [44]	83	Fecal incontinence	Yes	No	Yes	Yes	R-VF	18		Yes	Surgical fistula repair
Matthews et al., 2020 [45]	66	Constipation and persistent vaginal bulge	Yes	No	Yes	Yes	R-VF	60	5	Yes	Fistula repair, ileostomy, and hysterectomy
Yan et al., 2020 [46]	91	Urinary incontinence	Yes	No	Yes	Yes	Gellhorn shelf pessary in urinary bladder	10	2	Yes	Migrated pessary removal
Pereira et al., 2020 [47]	72	Vaginal bleeding	Yes	Yes	No	No	VPI	6	3	No	Pessary removal (LA)
Rieben et Annette, 2020 [48]	58	Vaginal bleeding	Yes	No	No	No	Arterial vaginal bleeding	0.75	0.057	No	Surgery to control hemorrhage
Mendelson et al., 2021 [49]	99	Vaginal discharge	Yes	No	Yes	Yes	R-VF	4.5	3	Yes	Surgical treatment rejected due to patient’s characteristics
Mendelson et al., 2021 [49]	80	Vaginal discharge	Yes	No	No	Yes	R-VF	4.5	3	Yes	Surgical fistula repair
Mendelson et al., 2021 [49]	86	Vaginal discharge	Yes	No	No	Yes	R-VF	12	Long time	Yes	Surgical fistula repair
C Tan et J Faiz, 2021 [50]	81	Vaginal bleeding	Yes	No	No	No	Vaginal wall ulcer, squamous cell carcinoma	24	4	Yes	Vaginal hysterectomy and bilateral salpingo-oophorectomy
C Tan et J Faiz, 2021 [50]	72	Vaginal ulceration	Yes	No	No	No	Vaginal ulcer resulting in cancer 5 years after	-	15	Yes	Vaginal hysterectomy, bilateral salpingo-oophorectomy
C Tan et J Faiz, 2021 [50]	98	Vaginal bleeding	Yes	No	No	No	Vaginal squamous cell carcinoma	6	7	No	Conservative treatment due to patient’s characteristics
Goodwin et al., 2021 [51]	81	Continuous urinary leakage	No	No	Yes	Yes	Pessary eroded into bladder near trigone	24	5	Yes	Ureteroneocystostomy with psoas hitch and vesicovaginal fistula
Bae et al., 2021 [52]	79	Vaginal spotting	Yes	Yes	No	No	Adhesions and vaginal entrapment of pessary	72	11	Yes	Surgical removal (GA)
Bae et al., 2021 [52]	80	Lower abdominal pain	No	Yes	No	No	VPI	81	7	Yes	Surgical removal (GA)
Bhat et al., 2021 [53]	81	Urine frequency and urgency	Yes	No	Yes	Yes	V-V F	6	6	No	Conservative treatment due to patient’s characteristics
Amshel, 2005 [54]	86	Gas and stool passage from the vagina	Yes	No	Yes	Yes	R-VF	180	15	Yes	Transanal removal of the pessary, colostomy
Tarr, 2008 [55]	82	Passing stool through the vagina	Yes	No	Yes	Yes	R-VF	48	5	Yes	Vaginal hysterectomy, fistula repair
Torbey, 2014 [56]	75	Passing discharge through the vagina	Yes	No	Yes	Yes	R-VF	4	3	No	Colostomy while waiting for vaginal and RCF repair
Ozuner, 2015 [57]	82	Stress urinary. Vaginal bleeding	Yes	No	Yes	Yes	R-VF	60	5	Yes	1st attempt: Transanal repair, laparoscopy. 2nd attempt: transanal repair, low anterior resection.
Gordon, 2015 [58]	88	Abdominal Pain	No	No	Yes	Yes	R-VF	-	-	Yes	Colostomy, transrectal removal, mini-laparotomy
Gordon, 2015 [58]	64	Urinary retention	No	No	Yes	Yes	R-VF	0,5	-	No	Laparoscopic Harmann colostomy, vaginal hysterectomy
Reinsenauer, 2017 [59]	87	Stool leaking through the vagina	Yes	No	Yes	Yes	R-VF	6	16	No	Ileostomy, vaginal hysterectomy
Christopher, 2017 [60]	61	Stool leaking through the vagina	Yes	No	No	Yes	R-VF	2	0.16	No	Resection of the RVF, hysterectomy
Wilhelm, 2020 [61]	81	Fecal discharge through the vagina	Yes	No	Yes	Yes	R-VF	-	-	Yes	Loop ileostomy, fistula closure, LeFort colpocleisis, ileostomy closure
Wilhelm, 2020 [61]	87	Fecal discharge through the vagina	Yes	No	Yes	Yes	R-VF	-	-	Yes	Loop ileostomy, fistula closure, LeFort colpocleisis, vaginal hysterectomy, ostomy closure
Wilhelm, 2020 [61]	83	Fecal discharge through the vagina	Yes	No	Yes	Yes	R-VF	-	-	Yes	Loop ileostomy, fistula closure, LeFort colpocleisis, vaginal hysterectomy, ostomy closure
Calles et al., 2023	82	Asymptomatic	No	Yes	No	No	VPI	6	6	No	Vaginoscopic removal
Calles et al., 2023	83	Vaginal discharge	Yes	No	No	Yes	R-VF	1	3	No	Fistula repair

Abbreviations: yr: years; mo: months; GA: general anesthesia; LA: local anesthesia; V-VF: vesicovaginal fistula; R-VF: rectovaginal fistula; VPI: vaginal pessary incarceration or entrapment; CPI: cervical pessary incarceration or entrapment; w: weeks; d: days; VHT: vaginal hysterectomy.

## Data Availability

**The** data presented in this manuscript are available from the corresponding authors on reasonable request.

## Supplementary Materials

The following supporting information can be downloaded at: https://www.mdpi.com/article/10.3390/jpm13071056/s1, Video S1: Safe vaginoscopic release of a ring pessary incarceration.
